# Multi-Branch Network with Multi-Feature Enhancement for Improving the Generalization of Facial Forgery Detection

**DOI:** 10.3390/e27050545

**Published:** 2025-05-21

**Authors:** Siyu Meng, Quange Tan, Qianli Zhou, Rong Wang

**Affiliations:** 1 College of Information and Cyber Security, People’s Public Security University of China, Beijing 100038, China; 13069390181@163.com (S.M.); tanquange@126.com (Q.T.); 2 Beijing Public Security Bureau, Beijing 100038, China; 13331112522@189.cn

**Keywords:** multi-branch, multi-feature, facial forgery detection, deepfake facial datasets

## Abstract

The rapid development of deepfake facial technology has led to facial fraud, posing a significant threat to social security. With the advent of diffusion models, the realism of forged facial images has increased, making detection increasingly challenging. However, the existing detection methods primarily focus on identifying facial forgeries generated by generative adversarial networks; they may struggle to generalize when faced with novel forgery techniques like diffusion models. To address this challenge, a multi-branch network with multi-feature enhancement (M2EH) model for improving the generalization of facial forgery detection is proposed in this paper. First, a multi-branch network is constructed, wherein diverse features are extracted through the three parallel branches of the network, allowing for extensive analysis into the subtle traces of facial forgeries. Then, an adaptive feature concatenation mechanism is proposed to integrate the diverse features extracted from the three branches, obtaining the effective fused representation by optimizing the weights of each feature channel. To further enhance the facial forgery detection ability, spatial pyramid pooling is introduced into the classifier to augment the fused features. Finally, independent loss functions are designed for each branch to ensure the effective learning of specific features while promoting collaborative optimization of the model through the overall loss function. Additionally, to improve model adaptability, a large-scale deepfake facial dataset, HybridGenFace, is built, which includes counterfeit images generated by both generative adversarial networks and diffusion models, addressing the limitations of existing datasets concerning a single forgery type. Experimental results show that M2EH outperforms most of the existing methods on various deepfake datasets.

## 1. Introduction

With the rapid development of deepfake technology, facial forgery generation techniques [1] are constantly emerging, making the generated images and videos increasingly realistic. This may lead to unethical behaviors such as identity theft and online fraud, thereby threatening social security. Therefore, developing effective deepfake facial detection methods is particularly important. Most existing deepfake facial detection methods are developed based on datasets generated by generative adversarial networks (GANs) [2] such as FaceForensics++ (FF++) [3] and Celeb-DF [4], and numerous benchmark datasets [3,4,5,6,7,8,9,10,11,12] have been proposed for this task. Although these methods have achieved impressive results in detection performance, several issues remain. With the rise of diffusion models (DMs) [13], some deepfake algorithms [14,15,16] have begun to generate fake facial images based on DMs. DMs not only provide a performance comparable or even superior to GANs but also support powerful text-to-image generation capabilities, enabling the creation of highly realistic images. Although significant progress has been made in the detection of facial images generated by GANs, Huang et al. [17] have demonstrated that existing facial forgery detectors, despite claiming to possess generalizability, are, in fact, unable to effectively identify facial images generated by different DM algorithms. This indicates the fundamental difference between images generated by DMs and those produced by GANs. Therefore, there is a need to develop facial forgery detection methods with greater generalization to address fake facial images generated by different generative models.

Most existing facial forgery detection methods predominantly rely on convolutional neural networks (CNNs), which are designed to capture local features. MesoNet [18] leverages CNNs to extract mesoscopic attributes for identifying facial images generated by techniques like deepfake and Face2Face. Nguyen et al. [19] integrate VGG-19 and capsule networks to enhance generalization by learning complex hierarchical representations. A multi-task network [20] simultaneously detects and localizes manipulated regions in images and videos, employing minimal data for fine-tuning to improve detection accuracy. However, the inherent limitation of CNNs’ local receptive field means that these purely CNNs-based methods may acquire specific inductive biases, thereby restricting their generalization capabilities. To address this, recent studies have increasingly adopted multi-branch network architectures for extracting diverse features to mine forgery traces, aiming to achieve more accurate detection and improved generalization. Dagar et al. [21] propose a dual-branch model that combines manually designed feature noise with conventional features, utilizing edge supervision loss and feature augmentation to effectively localize image manipulations and demonstrate superior performance on diverse datasets. Guo et al. [22] introduce a space–frequency interactive convolution with a multi-channel constrained separable convolution, using two branches to generate features that are interactively fused in three stages to model deepfake manipulation clues, replacing standard convolutions without altering the network structure. Khormali et al. [23] propose an end-to-end deepfake detection framework using a transformer to model local image features and global pixel relationships. The framework employs a dual-branch multi-stream transformer block to capture hidden perturbation traces across different forgery scales. The multi-branch network can integrate different neural network architectures to capture diverse features, thereby enhancing the generalization ability of detection. Thus, this paper also adopts a multi-branch network structure.

In summary, this paper proposes a multi-branch network with multi-feature enhancement (M2EH) for improving the generalization of facial forgery detection. First, a multi-branch network is constructed to extract various features through its three parallel branches. The texture feature extraction branch focuses on uncovering complex textures and structural anomalies in deepfake facial images, the fine-grained feature extraction branch is adept at capturing subtle features, and the multi-scale feature extraction branch dynamically adjusts the focus area through the additive attention mechanism to enhance the model’s adaptability to both local and global features. After extracting multiple features through the three branches, the adaptive feature concatenation mechanism is proposed to integrate these features by automatically adjusting the weights of each feature channel based on the characteristics of the input features, thereby achieving more efficient fused feature representation. Additionally, since the forged regions generated by different forgery methods vary, spatial pyramid pooling is introduced into the classifier to enhance the fused features, further improving the expressive capability of the fused features to enhance the classifier’s discriminative ability. Finally, independent loss functions are designed for each branch, aimed at ensuring that each branch effectively learns its specific features while promoting collaborative optimization of the model through the combination of the overall loss function. Notably, current deepfake facial datasets are primarily generated by GANs, while those produced by DMs are limited as most DMs-generated datasets focus on general images with little research on facial datasets. Consequently, most detection methods are designed for GANs-generated facial images. To address this issue, a large-scale dataset, HybridGenFace (HGF), is built, aimed at providing a more comprehensive dataset to evaluate and enhance the generalizability of facial forgery detection methods. This dataset contains over two million forged facial images generated by both GANs and DMs. We collect original images from the CelebA and FFHQ datasets and employ five different forgery algorithms to generate forged facial images, providing three different resolutions to simulate the real-world quality of encountered forged facial images. Experimental results indicate that our M2EH achieves significant performance across multiple deepfake facial datasets as well as our self-built HGF dataset.

The contributions of this paper can be summarized as follows:

(1) A multi-branch network is constructed to extract various features through its three parallel branches, delving deeply into the subtle traces of forgery present in facial images, thereby improving the generalization of facial forgery detection.
(2) The adaptive feature concatenation mechanism is proposed to integrate diverse features extracted from the three parallel branches, which dynamically adjusts the weights of each feature channel, allowing the model to focus on the most relevant features.
(3) Spatial pyramid pooling is introduced into the classifier to enhance the expressive power of fused features, enabling the model to better capture the spatial hierarchies and variations within the input features, which is crucial for effective deepfake detection.
(4) The independent loss functions are designed for each branch, aimed at ensuring that each branch effectively learns its specific features, while the combination of these functions through the overall loss function promotes collaborative optimization of the model.
(5) A large-scale dataset, HybridGenFace, is built, which including over two million synthetic facial images generated by GANs and DMs, addressing the gap in existing datasets that predominantly focus on a single generation type, thereby providing a comprehensive dataset for training and evaluating facial forgery detection methods.

## 2. Related Work

### 2.1. Deepfake Face Generation

In 2017, a user named “Deepfake” posted a face-swapped video on Reddit, marking the rise of deepfake technologies [24]. The term “deepfake” became synonymous with this technology, leading to increased scholarly research. Research expanded from simple face swapping to multi-attribute and multi-modal forgery techniques, enhancing the complexity of deepfake technologies. Currently, the two main deepfake generative models are GANs [2] and DMs [13]. GANs [2] consist of a generator and a discriminator that interact to create realistic forged images. StarGAN [25] enables face attribute transformation in a single model, while StyleGAN [26] separates high-level attributes for intuitive control over generated faces. DRFI GAN [27] combines VGG-16 and ResNet-50 to effectively generate realistic facial images. ClipFaceShop [28] transfers clipart attributes to photos, and HyperEditor [29] adjusts StyleGAN2 weights for real face editing. DMs [13] have advanced image generation, utilizing the UNet [30] architecture and the denoising process for high-quality outputs. DiffFace [14] and DCFace [15] focus on identity and style control in image generation. Collaborative diffusion [16] enhances multi-modal generation, while DreamBooth [31] improves thematic consistency. As forgery algorithms evolve, the need for effective detectors to distinguish real from fake images becomes critical.

### 2.2. Existing Deepfake Facial Datasets

The effectiveness of deepfake detection methods depends on the training data and evaluation process, leading to a growing need for large-scale deepfake datasets [4]. The UADFV dataset [5] includes 49 real and 49 deepfake videos generated via FakeAPP. The deepfake-TIMIT dataset [6], based on Vid-TIMIT, contains 320 real videos and 640 deepfake videos created using Faceswap-GAN, divided into low-quality and high-quality subsets. The FaceForensics++ [3] dataset comprises 1000 real YouTube videos and 4000 deepfake videos produced via four techniques (deepfakes, Face2Face, Faceswap, and NeuralTexture). The Google/Jigsaw Deepfake Detection Dataset [7] features 3068 deepfake videos from 363 raw videos by 28 actors of various demographics. The Facebook Deepfake Detection Challenge (DFDC) Dataset [8] includes 4113 deepfake videos based on 1131 original videos from 66 individuals. The WildDeepfake dataset [9] was created by searching for “deepfake” online, resulting in 707 videos after filtering, which yielded 7314 facial sequences and over 1 million images. The Celeb-DF dataset [4] consists of 590 real videos and 5639 deepfake videos. The ForgeryNet dataset [10] employs 15 forgery methods, generating 2.9 million images and 221,247 videos with extensive annotations. The datasets introduced earlier are mostly generated based on GANs, while the GFW dataset [11] and Diff dataset [12] are generated based on DMs. The GFW dataset [11] contains 15,076 forged facial images generated by various algorithms, and the Diff dataset [12] includes over 500,000 images synthesized under specific conditions. The aforementioned datasets include only GANs or DMs as the types of forgery, and there has yet to be a dataset that simultaneously utilizes both types of forgery. Furthermore, datasets based on DMs primarily generate general images, while those specifically designed for generating facial images are relatively scarce. To address these issues, we built a large-scale dataset, the HybridGenFace (HGF) dataset, comprising mixed facial forgery images generated by GANs and DMs. Five different generative algorithms are employed to synthesize over 2 million images, providing three resolutions. This makes the HGF dataset of significant reference value in the research of facial forgery detection methods.

### 2.3. Facial Forgery Detection

With the advancement of deepfake technologies, facial generation algorithms are increasingly focused on creating hyper-realistic images with finer textures and more natural expressions, which enhance the fidelity of forged faces and present significant challenges for deepfake detection. Facial forgery detection methods must achieve higher accuracy and generalization to detect various forgeries. MesoNet [18] uses CNNs to extract mesoscopic attributes for identifying facial images generated by deepfake and Face2Face techniques. Nguyen et al. [19] combine VGG-19 and capsule networks to improve generalization by learning complex layer representations. CViT [32] merges CNNs and vision transformer, where CNNs extract features and vision transformer classifies them using attention mechanisms. Multi-task networks [20] detect and locate manipulated areas in images and videos, fine-tuning with minimal data to enhance detection accuracy. Zhao et al. [33] propose a multi-attention deepfake detection network that includes multiple spatial attention heads and feature aggregation mechanisms, introducing region independence loss and attention-guided data augmentation to improve generalization. Most existing deepfake detection methods rely on traditional CNNs, which are constrained by their fixed receptive fields and thus inevitably introduce specific inductive biases. In recent years, related research has gradually shifted toward adopting multi-branch network architectures to mine forgery traces from multiple dimensions, aiming to achieve more accurate detection. Dagar et al. [21] propose a dual-branch model that integrates manually designed feature noise with conventional features to effectively localize image manipulations by employing edge supervision loss and feature augmentation, achieving superior performance on various datasets. Guo et al. [22] propose a space–frequency interactive convolution with a multi-channel constrained separable convolution, which uses two branches to generate features, which interact and fuse in three stages to model deepfake manipulation clues, replacing vanilla convolutions without altering network structures. Khormali et al. [23] propose an end-to-end deepfake detection framework using a transformer to learn local image features and global pixel relationships, featuring a multi-stream transformer block for dual-branch processing to capture hidden perturbation traces across forgery scales. Luo et al. [34] introduce a multi-branch framework that leverages high-frequency noise analysis and integrates three core components. These include a multi-scale high-frequency feature extraction module designed to capture forgery traces across diverse scales, a residual-guided spatial attention mechanism that sharpens the focus of RGB features on manipulated regions, and a cross-modality attention strategy that fosters synergistic interactions between RGB and high-frequency modalities to strengthen generalization capabilities. Miao et al. [35] introduce a dual-branch architecture that integrates CNNs and a transformer to hierarchically amalgamate local details and global contextual features, while strategically leveraging frequency domain information to facilitate cross-branch complementarity and enable multi-level frequency artifact exploration. To ensure the reliability of deepfake detection methods, it is essential to make them independent of generation types as different generation types may present varying qualities and appearances, which could limit the widespread application of detectors. Therefore, distinct from the aforementioned dual-branch methods, this paper proposes a three-branch network that captures diversified features and uncovers subtle forgery artifacts to improve the generalization of facial forgery detection.

## 3. Method

**Figure 1:**
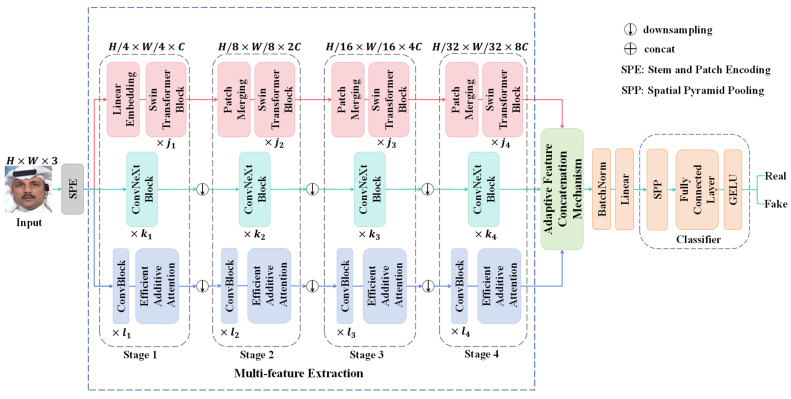
The structure of the proposed multi-branch network with multi-feature enhancement.

In this paper, a multi-branch network with multi-feature enhancement for improving the generalization of facial forgery detection is proposed, as illustrated in Figure 1. The M2EH primarily consists of a multi-branch network with multi-feature extraction, an adaptive feature concatenation mechanism, and a classifier based on spatial pyramid pooling. Initially, the input image is processed through the stem and patch encoding (SPE) layer to extract low-level features $F\in {\mathbb{R}}^{H/4\times W/4\times C}$. Subsequently, $F\in {\mathbb{R}}^{H/4\times W/4\times C}$ are fed into the multi-feature extraction to effectively extract diverse features by leveraging the multi-branch network. The texture feature extraction branch utilizes Swin transformer blocks, leveraging the moving window mechanism of the Swin transformer [36] to capture long-range dependencies, which is essential for detecting complex textures and structural anomalies in deepfake facial images. Meanwhile, the fine-grained feature extraction branch utilizes ConvNeXt blocks, effectively capturing subtle features in facial images due to exceptional feature extraction capabilities and robust representation learning abilities of ConvNeXt [37]. Additionally, the multi-scale feature extraction branch, utilizing ConvBlocks and efficient additive attention [38], further enhances the network’s adaptability to local and global features by dynamically adjusting the focus areas, making it particularly adept at handling common anomalies. After the multi-features are extracted through the four stages of the three branches, the features are then fed into the adaptive feature concatenation mechanism for fusion. The mechanism utilizes the weighted graph to automatically adjust the weights of each feature channel, thereby achieving more efficient fused representation. Finally, the fused features are enhanced by spatial pyramid pooling (SPP) [39] before being classified for the detection of facial forgery.

### 3.1. Multi-Branch Network with Multi-Feature Extraction

An input RGB image with a size of H×W×3 is processed through the SPE layer that employs non-overlapping 4×4 convolution along with layer normalization to achieve the feature map of H/4×W/4×C. Following this, the output feature maps are then fed into the multi-branch network, which comprises three parallel branches: the texture feature extraction branch, the fine-grained feature extraction branch, and the multi-scale feature extraction branch. Each branch is designed to extract distinct features, thereby delving deeper into the traces of facial forgery and enhancing the generalization of the model.

#### 3.1.1. Texture Feature Extraction Branch

**Figure 2:**
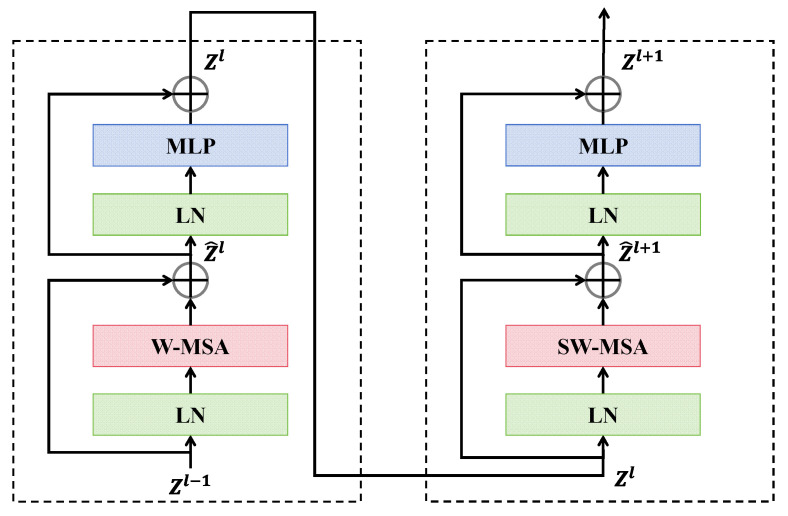
Two successive Swin transformer blocks.

The texture feature extraction branch, utilizing the Swin transformer, consists of four stages, each incorporating patch merging and Swin transformer blocks, except for the first stage, as illustrated in Figure 2. The Swin transformer is capable of capturing long-range dependencies based on the moving window mechanism, which is crucial for detecting complex textures and structural anomalies in deepfake facial images. The non-overlapping 4×4 patches from SPE are input into this branch, and then, linear embedding layers project these patches into the arbitrary dimension C, producing patch tokens $Z^{l-1}$, which are processed through the Swin transformer block. In this block, the self-attention operation is performed within the local window, with a window size set to M=7. The features after the W-MSA are then combined with $Z^{l-1}$ through the residual connection to obtain the processed output ${\hat{Z}}^{l}$. This output is further refined through norm layer and MLP, resulting in the feature map $Z^{l}$ for the first Swin transformer block:

(1) $$\Omega W-\mathrm{MSA}=4hwC^{2}+2M^{2}hwC$$

(2) $${\hat{Z}}^{l}=W-\mathrm{MSA}(\mathrm{LN}(Z^{l-1}))+Z^{l-1}$$

(3) $$Z^{l}=\mathrm{MLP}(\mathrm{LN}({\hat{Z}}^{l}))+{\hat{Z}}^{l}$$

The above process is repeated $j_{1}$ times for the feature maps $Z^{l-1}$ at “Stage 1”, resulting in a token count of H/4×W/4. Then, the extracted features from “Stage 1” are proceeded to patch merging in the second stage, which reduce the token count by combining adjacent 2×2 patches and pass them through the linear layer, resulting in a quarter of the tokens with the output dimension of 2C. These features are subsequently processed by $j_{2}$ Swin transformer blocks for further transformations, resulting in H/8×W/8 tokens. This procedure is repeated for “Stage 3” and “Stage 4” using $j_{3}$ and $j_{4}$ Swin transformer blocks, yielding feature maps with sizes H/16×W/16 and H/32×W/32, respectively. Ultimately, after completing the four stages, the branch outputs texture features $F_{\mathrm{texture}}$, which effectively helps the model in comprehending complex textures in deepfake facial images.

#### 3.1.2. Fine-Grained Feature Extraction Branch

Some deepfake facial algorithms, like FaceSwap, generate images with blurry boundaries, highlighting the importance of subtle information in deepfake detection. Thus, the fine-grained feature extraction branch is constructed, employing ConvNeXt blocks for subtle features extraction. ConvNeXt, known for its exceptional feature extraction capabilities and robust representation learning abilities, effectively captures subtle differences in various regions of deepfake facial images. Initially, the shallow texture feature $X^{l-1}$ from SPE is input into ConvNeXt Block at “Stage 1”, where it is first processed through a 7 × 7 convolutional layer, followed by normalization.

(4) $${\hat{X}}^{l}={\mathrm{Norm}(\mathrm{Conv}}_{7\times 7,C}(X^{l-1}))$$

The feature ${\hat{X}}^{l}$ is then passed through a 7 × 7 convolutional layer with the GELU activation function, and then the final 7 × 7 convolution layer, producing the output $X^{l}$.

(5) $$X^{l}={\mathrm{Conv}}_{7\times 7,C}(\mathrm{GELU}({\mathrm{Conv}}_{7\times 7,4C}({\hat{X}}^{l})))+X^{l-1}$$

By repeating these steps $k_{1}$ times, the output feature map for “Stage 1” is obtained. The output is then downsampled with the 7 × 7 convolution kernel before being proceeded to the next stage. This sequential process is repeated across three additional stages—“Stage 2”, “Stage 3”, and “Stage 4”—with $k_{2}$, $k_{3}$, and $k_{4}$ ConvNeXt blocks, respectively. The corresponding output dimensions for the feature maps are H/4×W/4×2C, H/8×W/8×4C, and H/16×W/16×8C. After completing four stages, the fine-grained feature extraction branch effectively captures subtle features $F_{\mathrm{fine}}$, significantly enhancing subtle forgeries, which is crucial for facial forgery detection.

#### 3.1.3. Multi-Scale Feature Extraction Branch

**Figure 3:**
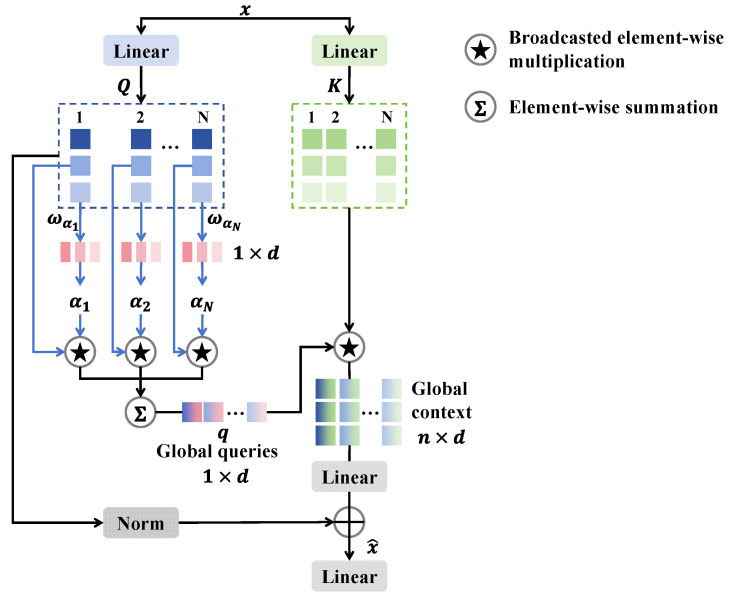
The structure of the efficient additive attention.

The multi-scale feature extraction branch is constructed to enhance the detail of various forged facial regions, as shown in Figure 3. This branch consists of four stages and three downsampling layers, each featuring several ConvBlocks and efficient additive attention. By utilizing efficient additive attention to dynamically adjust the weights of the feature maps, key regions are accentuated while background noise is mitigated, thereby augmenting the discriminative capacity of the features and enhancing the network’s adaptability to both local and global features, which makes it particularly proficient in addressing prevalent local anomalies associated with deepfakes. Initially, the feature map $Y_{i}$ from SPE is processed through a 3 × 3 convolutional layer and normalized. The processed features are then fed into two 1 × 1 convolutions with GeLU activation, followed by the residual connection for effective information flow. The ConvBlock is defined as follows:

(6) $${\hat{Y}}_{i}={\mathrm{Conv}}_{1}({\mathrm{Conv}}_{1,G}({\mathrm{DWConv}}_{\mathrm{BN}}(Y_{i})))+Y_{i}$$

where ${\mathrm{DWConv}}_{\mathrm{BN}}(\cdot )$ refers to depth-wise convolution followed by batch normalization, and ${\mathrm{Conv}}_{1,G}(\cdot )$ refers to point-wise convolution followed by GeLU. This process is repeated $l_{1}$ times to derive the feature $\hat{Y}$, which is then input to the efficient additive attention.

The efficient additive attention enhances performance by eliminating interactions between keys and values, focusing solely on the interactions between queries and keys through the use of linear projection layers for a more robust representation. The input feature $\hat{Y}$ is transformed into Q and K through matrices $W_{Q}$ and $W_{K}$, where $Q,K\in {\mathbb{R}}^{n\times d}$ and $W_{Q},W_{K}\in {\mathbb{R}}^{d\times d}$, where n denotes the token length and d denotes the dimensions of the embedding vector. Q is then multiplied by the learnable parameter $\omega_{\alpha }\in {\mathbb{R}}^{d}$ to generate the global attention query vector $\alpha \in {\mathbb{R}}^{n}$:

(7) $$\alpha =\frac{Q\cdot \omega_{\alpha }}{\sqrt{d}}$$

Next, the global attention query vector is processed by the element-wise product with Q to create the global query vector $q\in {\mathbb{R}}^{d}$, which is multiplied by K to produce global context $G\in {\mathbb{R}}^{n\times d}$. Then, the linear transformation is to be performed on G to obtain the learnable hidden token representation, which is added to the normalized query matrix, generating effective additive attention Y:

(8) $$Y=\mathrm{Norm}\left(Q\right)+\mathrm{Linear}\left(G\right)$$

Finally, Y is passed through the linear layer to yield the output feature maps for “Stage 1”. The features are then downsampled and passed through the subsequent stages, repeating this process for “Stage 2”, “Stage 3”, and “Stage 4”. After four stages of the multi-scale feature extraction branch, the multi-scale features $F_{\mathrm{ms}}$ are finally obtained.

### 3.2. Adaptive Feature Concatenation Mechanism

**Figure 4:**
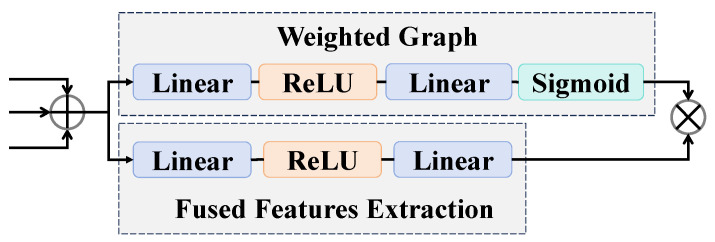
The structure of the adaptive feature concatenation mechanism.

Through the multi-branch network with multi-feature extraction, texture features $F_{\mathrm{texture}}$, fine-grained features $F_{\mathrm{fine}}$, and multi-scale features $F_{\mathrm{ms}}$, are obtained. Then, the adaptive feature concatenation mechanism is proposed to achieve the fusion of these three parallel features, as illustrated in Figure 4. The adaptive feature concatenation mechanism dynamically adjusts the contributions of three parallel features through the weighted graph, enhancing model adaptability and reducing redundant information interference. The adaptive feature concatenation mechanism first takes texture features $F_{\mathrm{texture}}$, fine-grained features $F_{\mathrm{fine}}$, and multi-scale features $F_{\mathrm{ms}}$ as inputs and then concatenates these three parallel features. Subsequently, the concatenated features are processed by two parallel networks: one is the weighted graph, which generates the weights for the features, and the other is the network that extracts the deep representations.

(9) $$F_{w}=\mathrm{Sigmoid}(\mathrm{Linear}(\mathrm{ReLU}(\mathrm{Linear}(F_{\mathrm{texture}}+F_{\mathrm{fine}}+F_{\mathrm{ms}}))))$$

(10) $$F_{f}=\mathrm{Linear}(\mathrm{ReLU}(\mathrm{Linear}(F_{\mathrm{texture}}+F_{\mathrm{fine}}+F_{\mathrm{ms}})))$$

Ultimately, the dynamic fusion process ultimately combines the features $F_{f}$ generated by the fused features extraction network with the weights $F_{w}$ produced by the weighted graph through element-wise multiplication, resulting in the final output $F_{\mathrm{Concat}}$.

(11) $$F_{\mathrm{Concat}}=F_{w}\;\cdot \;F_{f}$$

The above process yields dynamically adjusted fused features $F_{\mathrm{Concat}}$, which are subsequently input into the classifier.

### 3.3. Classifier Based on Spatial Pyramid Pooling

In order to enhance the fused features, after the entire feature extraction and fusion process, the SPP is introduced into the classifier. It pools the fused feature maps $F_{\mathrm{Concat}}$ into containers of different sizes and concatenates the resulting vectors to form the fixed-length output suitable for subsequent fully connected layers. This method avoids the drawbacks of resizing or cropping the images to the fixed-size input, while also being crucial for maintaining the spatial hierarchy between the concatenated feature maps $F_{\mathrm{Concat}}$ at different scales. The SPP layer with L levels can be defined as follows:

(12) $$\mathrm{SPPLayer}(F_{\mathrm{Concat}})={⊕}_{l=1}^{L}{\mathrm{pool}}_{l}(F_{\mathrm{Concat}})$$

Here, ⊕ denotes the concatenation operation, and pool(⋅) corresponds to the pooling operation at the *l*-th level, aimed at achieving average pooling across containers of different sizes. In this article, L is set to 4. The final output of SPP is the feature vector that contains the results of SPP at different levels. Finally, the detection results of binary classification (real or fake) are obtained through fully connected layers and GELU, achieving the detection of facial forgery.

### 3.4. Loss Functions

Cross-entropy loss. The fused features enhanced by SPP are fed into multiple fully connected layers to determine if the input image is real or fake, utilizing the cross-entropy loss $L_{cls}$:

(13) $$L_{cls}=y\log\hat{y}+(1-y)\log(1-\hat{y})$$

where y represents the true label value of the facial image, and $\hat{y}$ denotes the label value predicted by our method. When the facial image is manipulated, y is set to 1; otherwise, it is set to 0.

Branch loss: To incorporate the learning effects of each branch, separate loss functions are designed: $L_{1}$ for the texture feature extraction branch, $L_{2}$ for the fine-grained feature extraction branch, and $L_{3}$ for the multi-scale feature extraction branch. Each loss function is tailored to the unique characteristics of its respective branch, ensuring optimal learning of specific features. Formally, the branch loss is defined as follows:

(14) $$L_{1}=y_{1}\log{\hat{y}}_{1}+(1-y_{1})\log(1-{\hat{y}}_{1})$$

(15) $$L_{2}=y_{2}\log{\hat{y}}_{2}+(1-y_{2})\log(1-{\hat{y}}_{2})$$

(16) $$L_{3}=y_{3}\log{\hat{y}}_{3}+(1-y_{3})\log(1-{\hat{y}}_{3})$$

where $y_{i}$, i=1,2,3, is set to 1 if the facial image has been manipulated; otherwise, it is set to 0; and ${\hat{y}}_{i}$, i=1,2,3, denotes the predicted label by each branch. Combining Equations (13)–(16), the training objective can be written as follows:

(17) $$L=L_{cls}+\lambda_{1}L_{1}+\lambda_{2}L_{2}+\lambda_{3}L_{3}$$

where $\lambda_{1}$, $\lambda_{2}$, and $\lambda_{3}$ are the balancing hyperparameters. By default, we set $\lambda_{1}=0.001$, $\lambda_{2}=0.001$, and $\lambda_{3}=0.001$ [40].

## 4. HybridGenFace Dataset

The existing deepfake detection methods primarily focus on detecting facial forgery datasets generated by GANs, such as FF++ [3]. However, these methods may exhibit reduced generalization when applied to detecting forgeries generated by other methods, such as DMs. There is currently no dataset that mixes these two generative models. Therefore, a large-scale and challenging dataset, HybridGenFace (HGF), is built, aimed at providing a more comprehensive dataset to evaluate and enhance the generalization ability of facial forgery detection methods. The HybridGenFace dataset comprises both GANs and DMs as forgery types, including five forgery methods. As most detection methods are image-level based, the HGF dataset is image-based. It contains 716.16 K real facial images, and 2148.45 K forged facial images, with image quality categorized into three resolutions: 256×256, 512×512, and 1024×1024.

### 4.1. Data Collection and Processing

The original images are sourced from the CelebA and FFHQ datasets. CelebA is a large-scale facial dataset with over 200,000 celebrity images, covering various genders, ages, and ethnicities, and featuring rich pose variations and diverse background information. The FFHQ dataset contains 70,000 high-resolution facial images, with a wide range of ages, ethnicities, and backgrounds, and includes multiple facial attributes such as gender, skin color, expression, and hairstyle. To prevent the failure of forgery, the target face must be oriented directly forward. Therefore, we manually screen the images from two datasets to ensure the selection of high-quality facial images and to avoid instances of facial occlusion. We initially select 100,000 facial images from these two datasets as the original facial images for the HGF dataset. We then utilize the MTCNN [41] to identify the facial regions within each image and extract these regions, thereby minimizing interference from background elements and other factors.

### 4.2. Generation and Postprocessing

To generate a large-scale deepfake facial dataset, we adopt two prevailing types of synthesis: GANs and DMs. We select three GANs-based synthesis methods (BlendFace, E4S, VecGAN) and two DMs-based synthesis methods (DiffFace, DiffAE).

BlendFace [42] replaces the target image’s face with that of the source image using a blend mask predictor, effectively decoupling identity and attributes. E4S [43] performs face swapping in the latent space of a pre-trained StyleGAN through texture and shape swapping, utilizing FaceVid2Vid for pose and expression alignment. It estimates segmentation masks for both faces, processed by a mask-guided multi-scale encoder. Shape swapping stitches facial components while preserving lighting and skin features, with the final output being generated by StyleGAN. VecGAN [44] edits expressions by decomposing the latent space and calculating the shifts between target and source styles, maintaining image details through non-translation and cycle-translation paths. DiffFace [14] applies diffusion models to face-swapping, generating images with desired identities while preserving attributes and background. DiffAE [45] uses a learnable encoder and a diffusion probability model to encode images into semantically meaningful and random detail latent codes for reconstruction.

**Figure 5:**
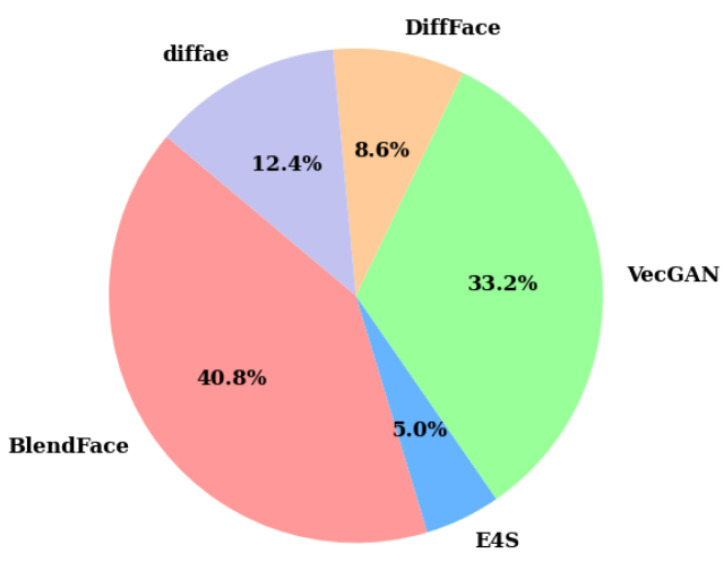
The proportion of images generated by each forgery algorithm in the HGF dataset.

To enhance the realism of the generated images, we generate counterfeit facial images at three different resolutions, akin to the significant quality degradation observed when images are uploaded to social media platforms. In summary, we show the statistics pertaining to images generated by the aforementioned operations in Table 1 and Figure 5. From Table 1, the HybridGenFace dataset contains 2148.45 K forged facial images generated by five forgery methods.

### 4.3. Comparison with Existing Deepfake Facial Datasets

#### 4.3.1. Statistical Analysis

The basic information of the HybridGenFace dataset in comparison with other facial forgery datasets is presented in Table 2, including the number of forgery methods and the quantities of real and forged image-level images contained within each dataset. It can be observed from Table 2 that although the number of forgery methods employed in our dataset is fewer than that of the ForgeryNet and Diff datasets, the HGF dataset integrates two currently predominant generative models simultaneously. Furthermore, most existing datasets based on DMs primarily focus on general images, with relatively few datasets dedicated to facial forgery. Notably, the number of forged images in HGF dataset exceeds that of the majority of facial forgery datasets, reaching over 2 million. The number of real facial images exceeds 700,000 in the HGF dataset. This is due to the facial forgery algorithms we use, such as DiffFace, which allows for the exchange of source images with multiple target images, thereby significantly increasing the quantity of real facial images. 

We also present the Mask-SSIM score, perceptual loss, and peak signal-to-noise ratio (PSNR) metrics in Table 3. Mask-SSIM is the SSIM score between the deepfake images and the original images. Perceptual loss focuses more on the perceptual quality of the images. We utilize the pre-trained VGG-19 network layers on ImageNet to compute the perceptual loss between real and fake facial feature maps. PSNR is a common metric for assessing image quality, measuring the similarity and differences between images. It is calculated using mean square error, effectively highlighting subtle distortions and noise, which aids in evaluating the quality of forged images. For both Mask-SSIM and PSNR, higher values indicate better image quality, while for perceptual loss, lower values signify superior image quality. Observations from Table 3 reveal that the HybridGenFace dataset demonstrates significant improvements across these metrics, indicating that the images within our dataset are closer to real images.

#### 4.3.2. Feature Space Distribution

**Figure 6:**
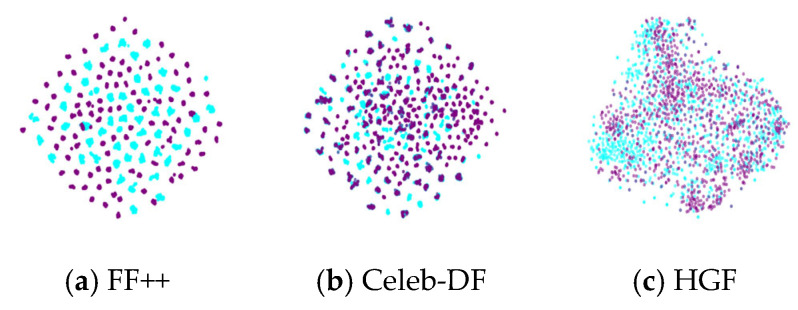
The comparison of feature distributions of three deepfake facial datasets.

We visualize the feature space of FF++, Celeb-DF, and HybridGenFace datasets, selecting 2000 real images and 2000 fake images from each dataset for visualization. We use the ResNetV2-101 network pre-trained on ImageNet [46] to extract features, and we use t-SNE [47] to reduce the dimensionality of the extracted features. Figure 6 shows the comparison of feature distributions of three deepfake datasets. The blue color represents real facial images, while the purple color denotes fake facial images. From Figure 6, it can be observed that the feature space distribution of the FF++ dataset exhibits a “clustering” phenomenon, which may be due to the fact that the dataset contains four different forgery methods, resulting in the images generated by each forgery method clustering together in the feature space. The data distribution of the Celeb-DF dataset is more concentrated, indicating that the samples in the Celeb-DF dataset have high similarity in the feature space, as the Celeb-DF dataset only uses one forgery method to generate all forged images, resulting in limited diversity in data distribution. The feature distribution of our HGF dataset appears more dispersed because we use different algorithms of GANs and DMs, resulting in greater differences in the features of the generated images. This difference in feature distribution is of great significance for the design and evaluation of deepfake detection methods. For dispersed datasets, detection methods may require stronger generalization ability to adapt to the diversity between different forged features.

#### 4.3.3. Detection Comparison

We conduct training of the capsule [48] using both the FF++ dataset and the HybridGenFace dataset to compare their generalization performance. Additionally, we utilize three deepfake facial datasets, namely, the Celeb-DF, WildDeepfake, and Diff datasets, for further evaluation. The HGF dataset is divided into training, validation, and testing sets in a ratio of 8:1:1 in order to maintain consistency with the partitioning of the FF++ dataset. The experimental results are presented in Table 4. As indicated in Table 4, the capsule trained on the HybridGenFace dataset demonstrates better generalization. In comparison to the capsule trained on the FF++ dataset, the testing results on other datasets are consistently higher than those obtained from the FF++ dataset. Notably, the capsule trained on the FF++ dataset exhibits relatively low AUC when tested on the HGF dataset, which further underscores the high difficulty of our HGF dataset.

## 5. Experiments

### 5.1. Experimental Settings

#### 5.1.1. Datasets

Training set: To ensure fair comparison with other detection methods, we select the FaceForensics++ dataset [3] as the training dataset, consistent with other detection approaches. We selected a high-quality (C23) dataset of FF++ to train the proposed M2EH. We follow the division of FF++, randomly selecting 720 training videos, 140 validation videos, and 140 test videos from every 1000 videos. We selected 80 frames from each video for training and validation, 40 frames for testing, and augmented the real images four times by repeated sampling to balance the number of real and fake samples.

Testing sets: We select datasets generated by either GANs or DMs forgery types and also use our own proposed dataset, HybridGenFace, as the testing datasets to evaluate the generalization of the proposed method. We select UADFV, DFDC, WildDeepfake, Celeb-DF-v1, Celeb-DF-v2, GID-DF, and GID-FF. GID-DF and GID-FF represent training on the other three forgery methods of FF++ but testing on deepfakes (DF) and Face2Face (FF), respectively. For datasets generated by DMs, we select Diff. In total, together with our HGF dataset, we use a total of nine datasets to verify the generalization of the proposed method. For these datasets, we randomly select 20,000 frames (images) of each of the real and fake images for evaluation.

#### 5.1.2. Implementation Details

We use RetinaFace [49] to crop the facial regions as inputs with size of 224×224. The modules of the texture feature extraction branch and fine-grained feature extraction branch are pre-trained on the ImageNet-1K, and the modules of multi-scale feature extraction branch are randomly initialized. The SPE layers are also randomly initialized. We use Adam for optimization, with a learning rate of 0.0001 and weight decay of 0.0001. The batch size is set to 16. Two variants are designed, including multi-branch network with multi-feature enhancement-T (M2EH-T), with C=96, and multi-branch network with multi-feature enhancement-S (M2EH-S), with C=96. The layer numbers of M2EH-T, corresponding to the Swin transformer block of the texture feature extraction branch, the ConvNeXt block of the fine-grained feature extraction branch, and the ConvBlock of the multi-scale feature extraction branch, are $\left\{j_{1},j_{2},j_{3},j_{4}\}=\{2,2,6,2\right\}$, $\left\{k_{1},k_{2},k_{3},k_{4}\}=\{3,3,9,3\right\}$, and $\left\{l_{1},l_{2},l_{3},l_{4}\}=\{4,4,12,6\right\}$, respectively. The layer numbers of M2EH-S are $\left\{j_{1},j_{2},j_{3},j_{4}\}=\{2,2,18,2\right\}$, $\left\{k_{1},k_{2},k_{3},k_{4}\}=\{3,3,27,3\right\}$, and $\left\{l_{1},l_{2},l_{3},l_{4}\}=\{4,4,36,6\right\}$. We train the complete network for 50 epochs and implement experiments with PyTorch and a NVIDIA GeForce RTX 4090.

#### 5.1.3. Evaluation Metrics

We use the accuracy (ACC) and area under the receiver operating characteristic curve (AUC) to evaluate the classification performance of the proposed method. Since our M2EH is an image-based model, we generate the predicted score using a single image at the image-level. The image-level detection methods are capable of not only detecting forged images but also identifying manipulated videos. Please note that the comparison results with state-of-the-art (SOTA) methods come from their paper; otherwise, we will point out which ones are the results of running their code.

### 5.2. In-Domain Evaluation

The experimental results with the SOTA methods on the HQ (C23) dataset of the FF++ dataset are shown in Table 5. Our method outperforms all compared methods on AUC. Our M2EH-S and M2EH-T outperform Xception [3] by approximately 4.94% and 4.89% on AUC, respectively. M2EH uses multiple stages and features to reveal forgery artifacts from different perspectives that Xception [3] cannot access. For the classical frequency-based methods, namely, SPSL [50], F^3^-Net [51], and F^2^Trans [52], M2EH-T is about 4.43%, 1.65%, and 0.51% higher on AUC, respectively. Although these methods use frequency domain information to mine the local texture information of deepfake facial images to supplement the global context information, our method fully mines the texture and fine-grained information and pays better attention to the relationships between pixels. In comparison with the two-branch networks, namely, HFI-Net [35] and PEL [53], M2EH-T exceeds by about 1.09% and 0.43% on AUC, respectively. Our method dynamically adjusts the contribution of each feature to fuse features. Although M2TR [54], LiSiam [55], SGF [56], and Local-Relation [57] have larger input sizes, our method can still achieve a better performance than them on AUC. These excellent performances result from the fine-grained feature extraction branch of M2EH, which learns invariant fine-grained forgery features. Our method is comparable to the performance of HRNet-18 [58], exceeding it by only 0.15% on AUC.

### 5.3. Cross-Domain Evaluation

To demonstrate the generalization of the proposed method, we experimentally validate it against other state-of-the-art methods on datasets generated by different forgery algorithms to assess its robustness and adaptability. More specifically, the models are trained on the FF++ (C23) dataset and tested on another nine datasets, as described in Section 5.1.1.

#### 5.3.1. Evaluation Experiments on CelebDF-V2, CelebDF-V1 and DFDC

The comparison on the CelebDF-V2, CelebDF-V1, and DFDC datasets with the SOTA methods is shown in Table 6, where the data of F^3^-Net [51] and M2TR [54] are sourced from F^2^Trans [52]. From Table 6, it can be seen that our method outperforms the other methods. Most previous methods, such as Xception [3], MaDD [33], and Face X-ray [60], exhibit significant performance degradation when testing other datasets. These methods fail to fully consider texture features and do not delve into fine-grained features; thus, they struggle to obtain robust generalized forgery features. Meanwhile, some methods based on frequency domain information, such as F^3^-Net [51], HRNet-18 [58], Face X-ray [60], and GFFD [34], fail to focus on texture features, resulting in a decline in their performance. MTD-Net [61] is based on CNNs architecture that easily learns image-specific inductive biases, resulting in overfitting on the training datasets and poor performance in detecting other datasets. Although M2TR [54] and Trans-FCA [62] utilize transformer to extract texture features, they still have shortcomings in mining fine-grained features, which limits their performance. Overall, M2EH achieves better performance on CelebDF-V2, CelebDF-V1, and DFDC datasets.

#### 5.3.2. Evaluation Experiments on GID-DF and GID-FF

We train the model with three manipulations of the FF++ (C23) dataset and test it with another manipulation. The comparison of the SOTA methods on GID-DF and GID-FF is shown in Table 7, where the data of F^3^-Net [51] and M2TR [54] are sourced from F^2^Trans [52], and the data of GocNet [63] and HIFE [64] are sourced from MH-FFNet [59]. In Table 7, it can be observed that the proposed M2EH is largely better than other forgery detection methods in terms of AUC. Although GID-DF and GID-FF have different manipulations, our method learns different features to detect forgeries and dynamically adjusts the feature channel.

#### 5.3.3. Evaluation Experiments on UADFV, WildDeepfake, Diff, and HybridGenFace

The comparison of the SOTA methods on the UADFV, WildDeepfake, Diff, and HybridGenFace datasets is shown in Table 8. Please note that we do not report quantitative AUC results for certain state-of-the-art deepfake detection methods because the code and model are not publicly available. From Table 8, it can be seen that our method outperforms the compared deepfake detection methods in testing different datasets. This suggests that our method does indeed possess better generalization. For the WildDeepfake dataset, forged videos are completely collected from the Internet, showing different scenes, faces, and activities, and they usually have low resolution. These fake videos are closer to the real-world dissemination environment, making it a challenging dataset. On this dataset, our M2EH-S outperforms M2TR [54] by approximately 11.05%, demonstrating that our multiple features exhibit more effective forgery artifacts. For our self-built HGF dataset, our method also achieves better performance, further proving that our method can not only detect forged facial images generated by GANs but can also effectively detect forged facial images generated by DMs with higher difficulty. In addition, the quality of different deepfake datasets can be evaluated by comparing the detection accuracy of the same detection method on different datasets. We calculate the average AUC scores of UADFV, WildDeepfake, Diff, and HybridGenFace among all comparison detection methods and report the results in the last row of Table 8. The results show that HybridGenFace has the lowest overall performance among the four datasets, indicating that our HybridGenFace dataset is the most challenging.

### 5.4. Visualization Experiments

#### 5.4.1. Visualization of Interpretable Decisions

Given the black-box nature of M2EH, interpretable decisions are crucial for trust and legal validation in deepfake detection. Therefore, we utilize Grad-CAM [71], an explainable AI technology, to visualize the model’s decisions. This technology identifies the parts of the facial image that are most important to the classification result by calculating the gradient of the classification score with respect to convolutional features. It focuses the model’s attention on specific areas of the image that play a key role in the final classification result. By visualizing these key areas, we can better understand the model’s decision-making logic, thereby enhancing the model’s transparency, reliability, and credibility.

**Figure 7:**
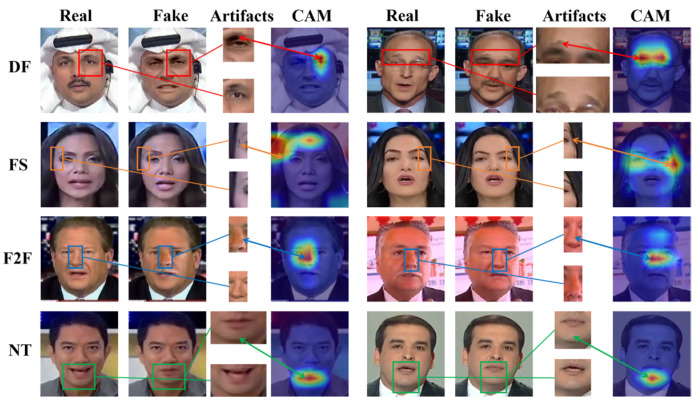
The visualization experiments of the proposed M2EH-T on the FF++ dataset.

**Figure 8:**
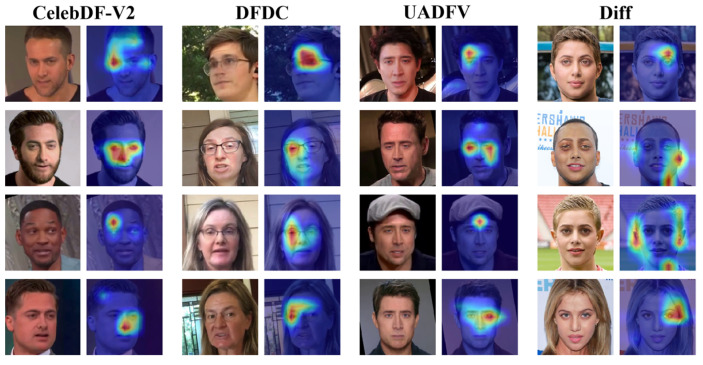
The visualization experiments of our method M2EH-T via attention maps.

Figure 7 shows the visual attention maps of images generated by four forgery methods of the FF++ (C23) dataset, including deepfakes (DF), Face2Face (F2F), FaceSwap (FS), and NeuralTextures (NT). The sections framed by rectangles in Figure 7 are areas containing artifacts or facial distortions. It can be seen that these boxed parts correspond to the regions of interest in the attention maps of M2EH-T, demonstrating how our method makes decisions by learning discriminative forgery features. Furthermore, F2F and NT forgery algorithms mainly target the subtle parts of the face, such as the nose and mouth. From the attention maps visualizing F2F and NT in Figure 7, it can be seen that M2EH-T focuses on the nose and mouth of the face, thereby successfully detecting forged images. During face swapping, the edges between the source image and the target image may appear blurry or represent unnatural transitions. Our method pays special attention to these mixed junctions in the fake facial images generated by FS and can effectively identify the unnatural transition regions in the forged images. These further enhance the interpretability of our method. In addition, we also generate visual attention maps of the regions of interest for M2EH-T on the CelebDF-V2, DFDC, UADFV, and Diff datasets, as shown in Figure 8. It can be seen that for different datasets, our method can still focus on artifacts in different facial regions, rather than being limited to fixed facial regions. This not only indicates that M2EH can capture the unique artifacts of different forgery algorithms but also demonstrates the flexibility of its decision-making logic through visualization results. Even trained on a single type of forgery dataset, it can dynamically identify key clues of other types of forgeries. Moreover, as shown in Figure 8, our method focuses on roughly the same regions for the same dataset. For example, in the DFDC dataset, our method specifically focuses on the area around the eyes. This is because the same dataset uses the same generation algorithm, resulting in roughly the same forged regions being generated. This further proves that the generalization of our method will not experience a significant drop when tested on datasets generated by different types of forgery, even if it was trained on a dataset generated by a specific type of forgery, due to the different areas of focus. Overall, the analysis of visualized attention maps shows that our method’s decisions are interpretable.

**Figure 9:**
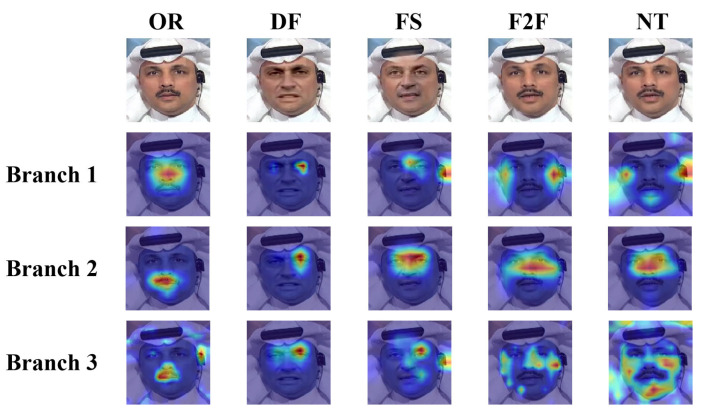
The visualization experiments of each branch.

In order to better understand the extraction capability of each branch, we visualize the attention maps of three branches for the facial images generated by four forgery methods in the FF++ dataset, including original images (OR), deepfakes (DF), Face2Face (F2F), FaceSwap (FS), and NeuralTextures (NT), as shown in Figure 9, where “Branch 1” refers to the texture feature extraction branch, “Branch 2” refers to the fine-grained feature extraction branch, and “Branch 3” refers to the multi-scale feature extraction branch. As can be seen in Figure 9, the texture feature extraction branch mainly focuses on texture information, such as details in the edge regions of the face. The fine-grained feature extraction branch pays more attention to capturing subtle traces, with its attention being concentrated on local regions to accurately locate forgery clues. The multi-scale feature extraction branch is responsible for extracting multi-scale features, and as can be seen from the Figure 9, the attention distribution of this branch is relatively scattered, which indicates that its feature extraction capability is weaker compared to that of the fine-grained feature extraction branch.

#### 5.4.2. Visualization of the Feature Distribution

**Figure 10:**
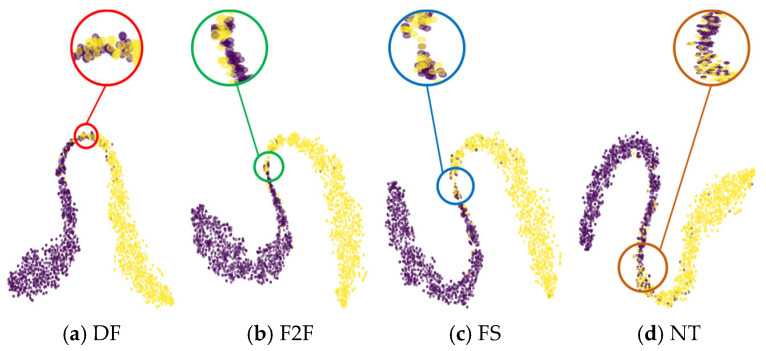
Feature space visualization of the M2EH-T.

In order to visually demonstrate the distribution of the proposed method in the feature space, we use t-SNE to visualize the selected samples from the FF++ dataset, as shown in Figure 10, where purple represents real facial images and yellow represents fake facial images. We select 1000 images for each of the two data types (real and fake) for visualization. It can be observed from Figure 10 that M2EH-T can separate real images from DF, F2F, FS, and NT images into two different clusters. We mark the overlapping parts in each visualized samples with circles. The overlap may be due to a misjudgment by M2EH. The overlap area between real and DF is relatively small, which allows M2EH to achieve higher accuracy on DF. However, there is more overlap between NT and real, which may be the reason for M2EH’s lower accuracy on NT.

### 5.5. Ablation Experiments

#### 5.5.1. Effectiveness of Different Modules

We analyze the impact of the three branches, adaptive feature concatenation mechanism, spatial pyramid pooling, and branch loss on the M2EH-T performance through experiments. The results are shown in Table 9. “Branch 1” refers to the texture feature extraction branch, “Branch 2” refers to the fine-grained feature extraction branch, “Branch 3” refers to the multi-scale feature extraction branch, and “Fuse” represents the adaptive feature concatenation mechanism. The M2EH-T is trained on the FF++ and tested on the CelebDF-V2 and DFDC datasets. The results in Table 9 indicate that the removal of any one of these modules will lead to a decline in model performance, particularly during cross-domain evaluation. When the multi-scale feature extraction branch is removed, the AUC on the CelebDF-V2 and DFDC datasets drops from 78.82% and 87.31% to 50.00% and 49.42%, respectively. This indicates that the multi-scale features extracted by our method are effective for the generalization of the model. Different forgery algorithms generate different forged facial regions, and multi-scale features can better model the relationships between pixels in different facial regions. Removing the texture feature extraction branch also significantly reduces the performance of cross-domain evaluation, and the decrease in the AUC of in-domain evaluation after removing the texture feature extraction branch is much greater than that after removing the multi-scale feature extraction branch. This suggests that texture features are more important for in-domain evaluation. Based on the three branches, the incorporation of SPP resulted in an approximate increase of 0.13% within the domain and 1.1% in the cross-domain. This demonstrates that SPP plays a crucial role in M2EH, significantly enhancing the generalization of the detection method. After removing the adaptive feature concatenation mechanism, the AUC for both in-domain and cross-domain evaluations decreased by approximately 0.11% and 0.22%, respectively. This indicates that the proposed mechanism is effective as it can adjust the weights of each feature, thereby facilitating better integration of the features from the three branches. Despite the addition of branch loss based on the three branches improving the in-domain and cross-domain AUC by approximately 0.06% and 0.26%, this indicates that branch loss does indeed play a particular role in M2EH. Removing the fine-grained feature extraction branch significantly reduces the performance of both in-domain and cross-domain evaluations, indicating that the fine-grained feature extraction branch plays a dominant role in the entire network. The fine-grained features can capture the relationships and dependencies between different regions in the facial images, enabling the model to more accurately distinguish between real and forged facial images. In summary, the results in Table 9 verify the effectiveness of the proposed modules and also show that the proposed M2EH has promising generalization potential.

#### 5.5.2. Effectiveness of the Number of Blocks of Each Branch

To determine the selection of the number of blocks for each branch of the network, the model is trained on the FF++ dataset and tested on the FF++, CelebDF-V2, and DFDC datasets. The experimental results are presented in Table 10. From Table 10, it can be observed that when the number of Swin transformer blocks are configured as {2, 2, 6, 2}, and when the numbers of the ConvNeXt blocks and ConvBlocks are set to {3, 3, 9, 3} and {4, 4, 12, 6}, respectively, the model achieves higher AUC values in both within-dataset and cross-dataset scenarios compared to the other two configurations. One configuration has the Swin transformer blocks, ConvNeXt blocks, and ConvBlocks set to {2, 2, 6, 2}, {3, 3, 27, 3}, and {4, 4, 36, 6}, respectively. Another configuration has Swin transformer blocks set to {2, 2, 18, 2}, while ConvNeXt blocks and ConvBlocks remain {3, 3, 9, 3} and {4, 4, 12, 6}, respectively. Furthermore, when the numbers of the Swin transformer blocks, ConvNeXt blocks, and ConvBlocks are set to {2, 2, 18, 2}, {3, 3, 27, 3}, and {4, 4, 36, 6}, respectively, the model’s AUC performance in both within-dataset and cross-dataset scenarios surpasses that of the two previously mentioned configurations. This may be attributed to the fact that when the number of blocks in a specific branch suddenly increases significantly, it can cause the model to overfit, resulting in a decline in the AUC value. Based on these findings, we focus on two optimal configuration combinations. The first combination includes Swin transformer blocks set to {2, 2, 6, 2}, ConvNeXt blocks to {3, 3, 9, 3}, and ConvBlocks to {4, 4, 12, 6}. The second combination involves Swin transformer blocks, ConvNeXt blocks, and ConvBlocks set to {2, 2, 18, 2}, {3, 3, 27, 3}, and {4, 4, 36, 6}, respectively.

#### 5.5.3. Effectiveness of the Hidden Dimension of the Adaptive Feature Concatenation Mechanism

Due to the involvement of the hidden dimension in the adaptive feature concatenation mechanism, this parameter, as the core hyperparameter of the feature fusion mechanism, directly regulates the model’s ability to dynamically integrate multi-branch features. A smaller hidden dimension may limit the network’s ability to model complex interaction relationships between different branch features, potentially leading to excessive information compression or insufficient feature representation. Conversely, a larger hidden dimension can enhance the model’s ability to integrate information, but it also significantly increases the number of parameters and the computational complexity, exacerbating the risk of overfitting. Therefore, to investigate the impact of the hidden dimension of the adaptive feature concatenation mechanism on the robustness of M2EH, the model is trained on the FF++ dataset and tested on the CelebDF-V2 and DFDC datasets. Given that the concatenated feature dimension is 3000, we set the hidden dimension to 1000 and 3000, respectively, to explore its impact on the effectiveness of the fusion mechanism. The experimental results are shown in Table 11. When the hidden dimension is increased from 1000 to 3000, the AUC in the intra-dataset experiment slightly increases from 99.75% to 99.78%, indicating that a higher hidden dimension slightly improves the model’s fitting ability on the same dataset. This may be because the higher hidden dimension enhances the selective attention capability in handling multi-branch features by increasing the degrees of freedom of non-linear transformations, enabling the model to more finely capture feature correlation patterns in the same data and thus improve the AUC. In cross-dataset scenarios, the AUC on CelebDF-V2 decreased from 79.54% to 78.37%, and the AUC on DFDC decreased from 88.92% to 88.01%. This may be because a larger hidden dimension is overly sensitive to specific noise or biases in the training data distribution and fails to effectively generalize to target datasets with significantly different distributions during cross-domain testing, leading to a decline in AUC. By setting the hidden dimension to 1000, which limits the complexity of intermediate representations, the model is forced to learn more universal feature interaction patterns. Although this sacrifices some in-dataset performance, it significantly enhances cross-domain performance, maintaining more stable robustness even with fewer parameters. Therefore, the hidden dimension of the adaptive feature concatenation mechanism is set to 1000 in this paper.

#### 5.5.4. Effectiveness of Different Weight Coefficients for the Branch Loss

To verify the impact of different training settings for the weight coefficients for three branch losses on the robustness of the model, M2EH is trained on the FF++ dataset and tested on the CelebDF-V2 and DFDC datasets. The experimental results are shown in Table 12, where Branch loss 1, Branch loss 2, and Branch loss 3 represent the loss weight coefficients for the texture feature extraction branch, the fine-grained feature extraction branch, and the multi-scale feature extraction branch, respectively. In the total loss function, the cross-entropy loss function is the core indicator by which to measure the difference between the model’s prediction results and the true labels, while the branch losses are used to encourage the model to achieve better performance on each branch. As can be seen from Table 12, the model’s AUC value is highest when all weight coefficients are 0.001 in both the within-dataset and cross-dataset scenarios. This indicates that the three branch losses play a positive role in model optimization, making the model’s robustness optimal. When the weight coefficients are 0.1, the AUC value decreases. This may be because excessively large weight coefficients cause the model to over-optimize branch losses, suppressing its learning of the global task. As a result, the model struggles to generalize to complex or unseen data, weakening its robustness. When the weight coefficients are 0.00001, the model’s AUC value is similar to the value without branch losses, indicating that such small weight coefficients have almost no impact on the optimization of branch losses. The model tends to optimize the cross-entropy loss, which, to some extent, weakens its optimization of branch losses. Therefore, the weight coefficients for the three branch losses are set to 0.001 in this paper.

## 6. Conclusions and Future Works

This paper proposes a multi-branch network with multi-feature enhancement (M2EH) for improving the generalization of facial forgery detection. A multi-branch network is constructed to extract various features through its three parallel branches, delving deeply into the subtle traces of forgery in facial images. After extracting multiple features through the three branches, the adaptive feature concatenation mechanism is proposed to integrate these features by automatically adjusting the weights of each feature channel, thereby achieving more efficient fused feature representation. Additionally, a spatial pyramid pool layer is introduced into the classifier to enhance the fused feature representation, which significantly improves the classifier’s recognition ability and the model’s robustness. Notably, independent loss functions are designed for each branch to ensure that each branch can effectively learn its specific features while promoting the model’s collaborative optimization through the combination of the overall loss function. Moreover, a deepfake facial dataset named HybridGenFace is built in this paper, which contains over two million synthetic facial images generated by GANs and DMs. This dataset is designed to provide a more comprehensive resource for evaluating and enhancing the generalization ability of facial forgery detection methods. Comparative experiments with existing detection methods demonstrate that the proposed method exhibits better generalization. Although the proposed method demonstrates promising performance, it still has several limitations, including potential shortcomings in dealing with complex forgery techniques that generate minimal artifacts and low-quality facial images with blurred features. While the self-built dataset is large in scale, it may not fully comprise the diverse variations in real-world scenarios such as illumination, pose, and occlusion. In the future, we will continue to explore more robust facial forgery detection methods. This will include developing robust models capable of effectively integrating multi-modal information, such as audio–visual cues and biological signals, to address complex forgery scenarios while maintaining high detection accuracy. Furthermore, we will also focus on researching solutions that are better suited to real-world facial forgery challenges.

## Figures and Tables

**Table 1:** Detailed statistics of the HybridGenFace dataset.

Type	Method	Images
GANs	BlendFace	876,261
	E4S	106,482
	VecGAN	714,000
DMs	DiffFace	185,385
	DiffAE	266,322
Total		2,148,450

**Table 2:** The basic information of the HybridGenFace dataset and existing deepfake facial datasets.

Dataset	Methods	Real	Fake
UADFV	1	17.3 K	17.3 K
Deepfake-TIMIT	2	34.0 K	34.0 K
FaceForensics++	4	509.9 K	1830.1 K
DFD	5	315.4 K	2242.7 K
DFDC	2	488.4 K	1783.3 K
WildDeepfake	-	440.5 K	739.6 K
Celeb-DF	1	225.4 K	2116.8 K
ForgeryNet	15	1438.2 K	1457.9 K
GFW	3	30 K	15.0 K
Diff	13	30 K	500 K
HybridGenFace (HGF)	5	716.16 K	2148.45 K

**Table 3:** The average Mask-SSIM score, perceptual loss, and PSNR of different deepfake datasets.

Dataset	UADFV	FF++	DFDC	Celeb-DF	GFW	HGF
Mask-SSIM↑	0.82	0.81	0.84	0.92	0.49	0.93
Perceptual Loss↓	0.73	0.67	0.63	0.59	0.98	0.53
PSNR↑	17.05	18.47	17.12	18.70	15.86	18.86

**Table 4:** The AUC (%) of the capsule trained on FF++ and HybridGenFace datasets.

Method	Training Datasets	Testing Datasets				
		FF++	HGF	Celeb-DF	WildDeepfake	Diff
Capsule	FF++	86.76	47.51	67.42	68.46	46.78
	HGF	87.50	70.62	67.95	70.03	50.09

**Table 5:** Comparison of the SOTA methods on the HQ dataset of FF++ dataset. The best results are marked in bold.

Methods	Input Size	AUC	ACC
Xception [3]	299	94.86	92.39
SPSL [50]	299	95.32	91.50
F^3^-Net [51]	299	98.10	97.52
HFI-Net [35]	384	98.66	95.12
LiSiam [55]	299	99.13	96.51
F^2^Trans-S [52]	224	99.18	96.09
F^2^Trans-B [52]	224	99.24	96.60
MaDD [33]	380	99.29	97.60
PEL [53]	320	99.32	97.63
MH-FFNet [59]	224	99.44	97.37
SGF [56]	320	99.34	**98.41**
Local-Relation [57]	229	99.46	97.59
M2TR [54]	320	99.51	97.93
HRNet-18 [58]	224	99.60	96.95
M2EH-T (Ours)	224	99.75	97.09
M2EH-S (Ours)	224	**99.80**	97.59

**Table 6:** Comparison of the SOTA methods on the CelebDF-V2, CelebDF-V1, and DFDC datasets. The best results are marked in bold.

Methods	Testing Dataset (AUC)		
	CelebDF-V2	CelebDF-V1	DFDC
M2TR [54]	65.17	68.57	69.94
Xception [3]	65.30	-	72.20
GocNet [63]	65.56	-	66.73
MaDD [33]	67.44	-	-
HIFE [64]	68.41	-	65.46
F^3^-Net [51]	68.69	63.57	67.45
MTD-Net [61]	70.12	-	-
HRNet-18 [58]	-	72.30	-
Face X-ray [60]	-	74.20	70.00
GFFD [34]	-	**79.40**	79.70
Trans-FCA [62]	78.57	-	-
M2EH-T (Ours)	79.54	77.46	**88.92**
M2EH-S (Ours)	**80.10**	76.89	88.78

**Table 7:** Comparison of the SOTA methods on GID-DF and GID-FF. The best results are marked in bold.

Methods	GID-DF		GID-FF	
	AUC	ACC	AUC	ACC
EfficientNet [65]	91.11	82.40	80.1	63.32
ForensicTransfer [66]	-	72.01	-	64.50
Multi-task [20]	-	70.30	-	58.74
MLDG [67]	91.82	84.21	77.10	63.46
LTW [68]	92.70	85.60	80.20	65.60
DCL [69]	94.90	87.70	82.93	68.40
M2TR [54]	94.91	81.07	76.99	55.71
F^3^-Net [51]	94.95	83.57	81.20	61.07
M2EH-T (Ours)	95.15	85.86	84.92	70.89
M2EH-S (Ours)	**95.23**	**86.50**	**86.66**	**70.91**

**Table 8:** Comparison of the SOTA methods on the UADFV, WildDeepfake, Diff, and HGF datasets. The best results are marked in bold.

Methods	Testing Dataset (AUC)			
	UADFV	WildDeepfake	Diff	HGF
Multi-task [20]	61.11	57.62	41.73	40.80
Capsule [48]	81.01	68.46	57.68	52.50
DCVit [32]	63.59	70.33	50.88	54.80
CNNDetection [70]	63.99	57.38	58.97	57.78
M2TR [54]	82.70	76.30	70.90	56.80
M2EH-S (Ours)	87.77	**87.35**	63.96	56.62
M2EH-T (Ours)	**90.75**	77.09	**72.58**	**59.42**
Avg	75.85	70.65	59.53	54.10

**Table 9:** Comparison of AUC with respect to the effects of different module combinations on the performance of the M2EH-T.

Branch 1	Branch 2	Branch3	Fuse	SPP	Branch Loss	FF++	CelebDF-V2	DFDC
√	√					95.60	50.00	49.42
√		√				57.42	50.56	52.33
	√	√				81.35	59.69	59.23
√	√	√				99.57	78.82	87.31
√	√	√	√			99.68	79.47	88.85
√	√	√		√		99.70	79.44	88.89
√	√	√			√	99.63	79.08	87.98
√	√	√	√	√	√	99.75	79.54	88.92

**Table 10:** Comparison of AUC with respect to the effect of the number of blocks of each branch.

Swin Transformer Block	ConvNeXt Block	ConvBlock	FF++	CelebDF-V2	DFDC
{2, 2, 6, 2}	{3, 3, 9, 3}	{4, 4, 12, 6}	99.75	79.54	88.92
{2, 2, 6, 2}	{3, 3, 27, 3}	{4, 4, 36, 6}	98.86	77.03	87.35
{2, 2, 18, 2}	{3, 3, 9, 3}	{4, 4, 12, 6}	98.66	78.17	86.22
{2, 2, 18, 2}	{3, 3, 27, 3}	{4, 4, 36, 6}	99.80	80.10	88.78

**Table 11:** Comparison of AUC with respect to the hidden dimension effect of the adaptive feature concatenation mechanism.

Hidden Dimension	Parameter	FF++	CelebDF-V2	DFDC
1000	125,619,785	99.75	79.54	88.92
3000	149,623,785	99.78	78.37	88.01

**Table 12:** Comparison of AUC with respect to the effects of different weight coefficients for the branch loss.

Branch Loss 1	Branch Loss 2	Branch Loss 3	FF++	CelebDF-V2	DFDC
0	0	0	99.71	79.37	88.03
0.1	0.1	0.1	99.61	78.72	87.91
0.001	0.001	0.001	99.75	79.54	88.92
0.00001	0.0001	0.0001	99.70	79.39	88.07

## Data Availability

The original contributions presented in this study are included in the article. Further inquiries can be directed to the corresponding author. The code is available at https://github.com/daisy-12138/M2EH (accessed on 16 March 2025).
